# Lipid Metabolism-Related Gene Markers Used for Prediction Prognosis, Immune Microenvironment, and Tumor Stage of Pancreatic Cancer

**DOI:** 10.1007/s10528-023-10457-y

**Published:** 2023-07-28

**Authors:** Yuan Shu, Haiqiang Huang, Minjie Gao, Wenjie Xu, Xiang Cao, Xiaoze Jia, Bo Deng

**Affiliations:** 1 The Second Clinical Medical College of Nanchang University, Nanchang, Jiangxi 330000 People’s Republic of China; 2 Departments of Endocrine, The First Hospital of Nanchang, Nanchang, Jiangxi 330008 People’s Republic of China; 3 Internet of Things Engineering, College of Wuxi University, Wuxi, Jiangsu 214000 People’s Republic of China

**Keywords:** Pancreatic cancer, Lipid metabolism, Immune microenvironment, Prognosis, Tumor stage

## Abstract

Recently, more and more evidence shows that lipid metabolism disorder has been observed in tumor, which impacts tumor cell proliferation, survival, invasion, metastasis, and response to the tumor microenvironment (TME) and tumor treatment. However, hitherto there has not been sufficient research to demonstrate the role of lipid metabolism in pancreatic cancer. This study contrives to get an insight into the relationship between the characteristics of lipid metabolism and pancreatic cancer. We collected samples of patients with pancreatic cancer from the Gene Expression Omnibus (GEO), the Therapeutically Applicable Research to Generate Effective Treatments (TARGET), and the International Cancer Genome Consortium (ICGC) databases. Firstly, we implemented univariate regression analysis to get prognosis-related lipid metabolism genes screened and a construction of protein–protein interaction (PPI) network ensued. Then, contingent on our screening results, we explored the molecular subtypes mediated by lipid metabolism-related genes and the correlated TME cell infiltration. Additionally, we studied the disparately expressed genes among disparate lipid metabolism subtypes and established a scoring model of lipid metabolism-related characteristics using the least absolute shrinkage and selection operator (LASSO) regression analysis. At last, we explored the relationship between the scoring model and disease prognosis, tumor stage, tumor microenvironment, and immunotherapy. Two subtypes, C1 and C2, were identified, and lipid metabolism-related genes were studied. The result indicated that the patients with subtype C2 have a significantly lower survival rate than that of the patients with subtype C1, and we found difference in abundance of different immune-infiltrating cells. The gene ontology (GO) and Kyoto Encyclopedia of Genes and Genomes (KEGG) pathway enrichment analyses revealed the association of these differentially expressed genes with functions and pathways related to lipid metabolism. Finally, we established a scoring model of lipid metabolism-related characteristics based on the disparately expressed genes. The results show that our scoring model have a substantial effect on forecasting the prognosis of patients with pancreatic cancer. The lipid metabolism model is an important biomarker of pancreatic cancer. Using the model, the relationship between disease prognosis, molecular subtypes, TME cell infiltration characteristics, and immunotherapy in pancreatic cancer patients could be explored.

**Supplementary Information:**

The online version contains supplementary material available at 10.1007/s10528-023-10457-y.

## Introduction

Pancreatic cancer is one of the major causes of death of cancer patients worldwide, and the number of patients has doubled in the past decades (Goral 2015; Klein 2021). Worldwide, the incidence of pancreatic cancer is expected to increase to 18.6 per 100000 by 2050, with an average annual growth rate of 1.1%, which means that pancreatic cancer will constitute a major public health burden (Gillen et al. 2010; Hu et al. 2021). Pancreatic cancer is now increasingly seen in younger patients. Because the disease has no early symptoms and can rapidly invade surrounding tissues and organs, it is one of the deadliest cancers (Zhao and Liu 2020). With the development of technology, diversified therapeutic approaches are used to treat pancreatic cancer, such as chemotherapy and radiation (Hudson et al. 2010; Ben-Josef et al. 2004). However, pancreatic cancer patients have an astonishingly destitute 5-year survival rate of as it were 5% (5). Chemotherapy and radiotherapy are the traditional treatment methods. However, their killing effects on tumor cells are not precise enough, and they are often accompanied by serious side effects (Biran et al. 1989). Therefore, targeted tumor gene therapy, which can precisely kill tumor cells, has attracted much attention (Zhu and Chen 2018). Due to the heterogeneity of tumors, no single molecular targeted therapy was used successfully for treatment (Chiorean and Coveler 2015). Therefore, it is extremely important to develop new biomarkers for tumor diagnosis and treatment.

Increasingly confirmations demonstrate that tumor will lead to significant dysregulation of lipid metabolism system. Lipid metabolism is accessible for tumor cells to get the energy, biofilm components, and signaling molecules, which are needed to proliferate, survive, invade, metastasize, and respond to tumor microenvironment impacts and tumor therapy (Bian et al. 2021). For example, colon tumor cells can obtain fatty acids from the adipocytes and use them (Wen et al. 2017). Increased fatty acid oxidation and synthesis of new lipids may also provide tumors with a survival advantage against chemotherapy and radiotherapy (Corn et al. 2020). Relevant findings suggest that limiting the availability of fatty acids can control tumor cell proliferation (Currie et al. 2013). Some genes related to lipid metabolism have been recorded during tumor treatment, such as beta-lactamase-like protein (LACTB) and sterol regulatory element-binding protein 1 (SREBP-1) (Keckesova et al. 2017; Guo et al. 2014). Therefore, a comprehensive analysis of genes correlated to lipid metabolism-related that affect tumor cell survival and metastasis can be utilized to instruct clinical decision-making and give more treatment choices for patients with pancreatic cancer. Moreover, abnormal expression of genes can also affect the prognosis for cancer patients (Győrffy et al. 2016). For example, aberrant PTPRO methylation in tumor tissues significantly impacts the prognosis of breast tumor (Li et al. 2014). Many researchers can predict tumor prognosis using gene risk assessment models (Wang et al. 2020; Jiang et al. 2020). We have chosen the same method to provide new insights into pancreatic tumor staging and prognosis prediction.

Tumor cell progression, metastasis, and the ability to resist treatment are related to the immune microenvironment (Wu et al. 2020).And the tumor microenvironment is formed by leukocytes, vascular endothelial cells, and fibroblasts (Yang and Zhang 2017). Fibroblasts can directly stimulate tumor cells through paracrine signaling and promote tumor cell proliferation (Augsten 2014; Jia et al. 2013). More and more studies have shown that the immune microenvironment of tumors is closely related to lipid metabolism status. For example, the downregulation of lipid catabolism and the upregulation of lipid hydroxylation may hinder bone remodeling and immune system development (Qian et al. 2021). Therefore, we can study the immune microenvironment through lipid metabolism-related genes, providing a direction for treating pancreatic tumor.

Considering that lipid metabolism-related genes offer great value in the diagnosis and treatment of pancreatic cancer, we have extracted 893 lipid metabolism-related genes guided by the literature. Then, we performed a univariate regression analysis and selected 83 genes, of which one gene was not detected in the pancreatic cancer samples obtained from the TARGET database, so we finally selected 82 lipid metabolism-related genes. We performed an unsupervised clustering analysis of the 82 genes and divided the patients into two subtypes. Kaplan–Meier generation plots indicated significant differences in survival between the two subtypes, and Tumor Immune Estimation Resource (TIMER) immune infiltration analysis also showed the distribution of immune cells differs somewhat between the two subtypes. We used two databases, TARGET and GSE62452, to perform the differential expression analysis on the genes of the two subtypes and screened out 50 genes with stable differential expression. Next, we established a lipid metabolism-related feature scoring model via the least absolute shrinkage and selection operator (LASSO) regression analysis. Based on the scores, we divided patients into high group and low group and found close correlation between high scores and poor patient outcomes. Further research showed the close connection between score and both tumor stage and the expression of immune checkpoint genes, which can guide clinical diagnosis and treatment of pancreatic cancer.

## Methodology

### Data Collection and Preprocessing

We used the GEO database (https://www.ncbi.nlm.nih.gov/geo/) and downloaded GSE57495 and GSE62452, which contained 63 pancreatic cancer samples and 65 pancreatic cancer samples, respectively. Additionally, we collected 181 pancreatic cancer samples from the Genomic Data Commons (GDC) Data Portal (https://portal.gdc.cancer.gov/) and 91 pancreatic cancer samples from the ICGC database (https://dcc.icgc.org/releases/current/Projects/PACA-AU/).

We removed the lines with zero gene expression in more than 50% of the samples and chose the average value of rows with duplicate gene names as the representative.

### Identification of Prognostic Genes Related to Lipid Metabolism

We collected 893 genes related to lipid metabolism from the Molecular Signatures database MSigDB (GSEA | MSigDB (gsea-msigdb.org)) and KEGG (KEGG: Kyoto Encyclopedia of Genes and Genomes) (Supplementary Table 1) and analyzed the expression of these lipid metabolism-related genes in GSE62452 via univariate cox regression analysis. In accordance with the results of analysis, 83 lipid-metabolism-related genes that can affect prognosis were screened and ensued the comparison of their expression in 181 samples obtained from GDC. Finally, we selected 82 genes for further analysis. Probability values (*p* values) less than 0.05 were considered statistically significant.

### Functional Analysis of Prognostic Lipid Metabolism-Related Genes

GO is a database aiming to establish a language vocabulary standard applicable to various species (Wilkerson and Hayes 2010). KEGG is a database that systematically analyzes gene functions and links genomic and functional information, including metabolic pathways, hierarchical classifications, and gene and genome databases (Kanehisa and Goto 2000). The R software package “clusterProfiler” was utilized to conduct the enrichment analysis. The minimum gene set was 5 and the maximum gene set was 5000. A *p*-value less than 0.05 and FDR less than 0.25 were statistically consistent.

### Constructing a Protein–Protein Interaction Network

We study the connection between the corresponding proteins of the selected 82 prognostic lipid metabolism-related genes via a construction of PPI network using STRING (STRING: functional protein association networks (string-db.org)), an online tool used to evaluate the information of the PPI network.

### Consensus Clustering Analysis of Genes Related to Lipid Metabolism

The “ConsensusClusterPlus” package in R software was used for cluster analysis, using agglomerative pam clustering with a 1-Spearman correlation distance and resampling 80% of the samples for 100 repetitions (Seiler et al. 2010). The optimal number of clusters was determined through the empirical cumulative distribution function (CDF) plot. R software package “stats” was used for principal component analysis (PCA) analysis to verify our previous results of the molecular model constructed based on the lipid metabolism genes. To determine the clinical significance of clusters generated by consensus clustering, we studied the correlation between molecular subtypes and survival outcomes (OS). The differences in OS between different subtypes were evaluated using Kaplan–Meier analysis, which was obtained from the “survival” packages (Rich et al. 2010). Additionally, we inferred the abundance of six common immune cells via TIMER, including B cells, CD4 + T cells, CD8 + T cells, macrophages, neutrophils, and DC (Li et al. 2017).

### Establishment of a Scoring Model for Lipid Metabolism-Related Characteristics

To further study the difference between lipid metabolism subtypes, we analyzed the differentially expressed genes of subtypes in GSE62452 and TARGET samples via the R software package “limma.” We used Venn diagram to illustrate intersections between the stable differentially expressed genes. Then, founded on the results we obtained, we performed LASSO regression analysis and a scoring model of lipid metabolism-related characteristics was established. Based on minimum *λ*, the weight of genes in the model was determined as the regression coefficient of each gene. Each sample risk score was calculated as ∑ coefficient * expression value. Additionally, according to the score of each sample, we chose the best intercept value to split the samples in TARGET into two groups, high group and low group. By means of Kaplan–Meier analysis, we explored the difference in survival status between the high group and the low group. Receiver operating characteristic (ROC) curves were used to analyze the sensitivity of our scoring model in predicting the prognosis of patients with pancreatic cancer. GSE62452 and ICGC samples were used to validate our model.

### Relationship Between Lipid Metabolism-Related Characteristic Scoring Model and Clinical Characteristics

Finally, we explored the connection between our characteristic scoring model correlated to lipid metabolism and different stages of pancreatic cancer. The difference in scores between samples at different stages was researched via a nonparametric test.

### Immunotherapy Targets and Lipid Metabolism-Related Characteristic Scoring Model

To study the relationship between our scores and the effect of immunotherapy, we selected seven common immunotherapy targets for research: CD276, TGFB1, TBFSF4, IL1A, TNFSF9, CD70, and SELP. We found that these seven targets were abnormally expressed in both high group and low group. A nonparametric test was performed for variance analysis.

### Statistical Analysis

R software (version 4.2.1) and its related software packages were utilized to process and analyze data, with a *p*-value less than 0.05, which was considered statistically significant.

## Results

### Identification and Pathway Analysis of Genes Correlated to Lipid Metabolism

To screen the lipid metabolism genes correlated to tumorigenesis and progression in pancreatic cancer, we compared the GSE62452 dataset with 893 lipid metabolism-related genes and obtained 83 lipid metabolism genes correlated to the prognosis of pancreatic cancer patients using univariate cox regression analyses. After examining the expression of the above screened genes in TARGET pancreatic cancer patient samples, we eliminated one gene that was not expressed. Finally, we screened 82 lipid metabolism genes for further strengthened research (Supplementary Fig. 1, Table 1).

**Fig. 1:**
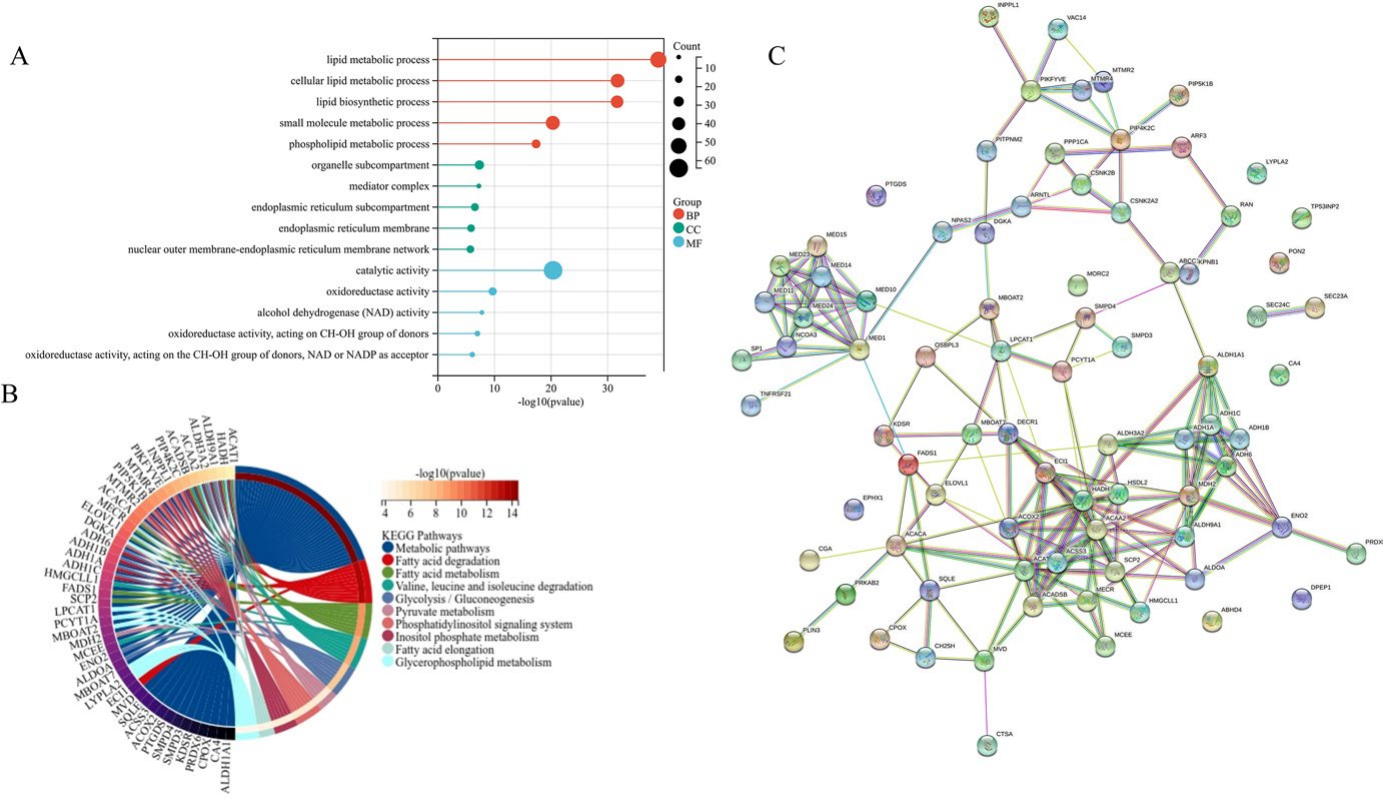
Basic information of Lipid metabolism-related genes. **A** GO enrichment analysis results of 82 lipid metabolism-related genes; **B** KEGG enrichment analysis results of 82 lipid metabolism-related genes; and **C** Protein–protein interaction network of 82 lipid metabolism-related genes

The correlation between the screened genes and lipid metabolism in pancreatic cancer cells was demonstrated by means of GO enrichment and KEGG analyses. The result of the former showed that, in terms of biological processes (BP), these genes were mainly enriched in lipid metabolic, cellular lipid metabolic, and lipid biosynthetic processes. In terms of molecular functions (MF), the biological molecules encoded by these genes showed catalytic activities, including oxidoreductase, alcohol dehydrogenase, and oxidoreductase activities. In terms of cellular components (CC), these genes were enriched among endoplasmic reticulum-related genes, responsible for lipid metabolism. The endoplasmic reticulum is involved in a diversity of lipid metabolism pathways, including cholesterol, phospholipids, and triglycerides (sterol esters and triacylglycerols). Synthetic lipids are dissociated in cells through vesicular and nonvesicular mechanisms and bind to corresponding target membranes. The result of the latter showed that these genes were considerably enriched in metabolic pathways, especially the degradation and metabolism of fatty acids and the metabolism of phospholipids, such as inositol phosphate and glycerophospholipids. Fatty acid synthesis has a significant effect on pancreatic cancer and various tumors, which can stimulate tumor survival and development (Sunami et al. 2017). Such results indicate that these genes are closely related to lipid metabolism (Fig. 1A, B).

Finally, we build a PPI network using STRING to identify the interactions of the screened genes. The results indicated that these screened genes showed a high degree of closeness and density and played an important role in regulating the PPI network (Fig. 1C). Then, we analyzed the PPI network of these 82 genes through the cytohubba plugin of cytoscape software to obtain a score for each gene calculated by 13 algorithms (Supplementary Table 1).

### Tumor Classification Model Founded on Lipid Metabolism-Related Genes and Its Validation

**Fig. 2:**
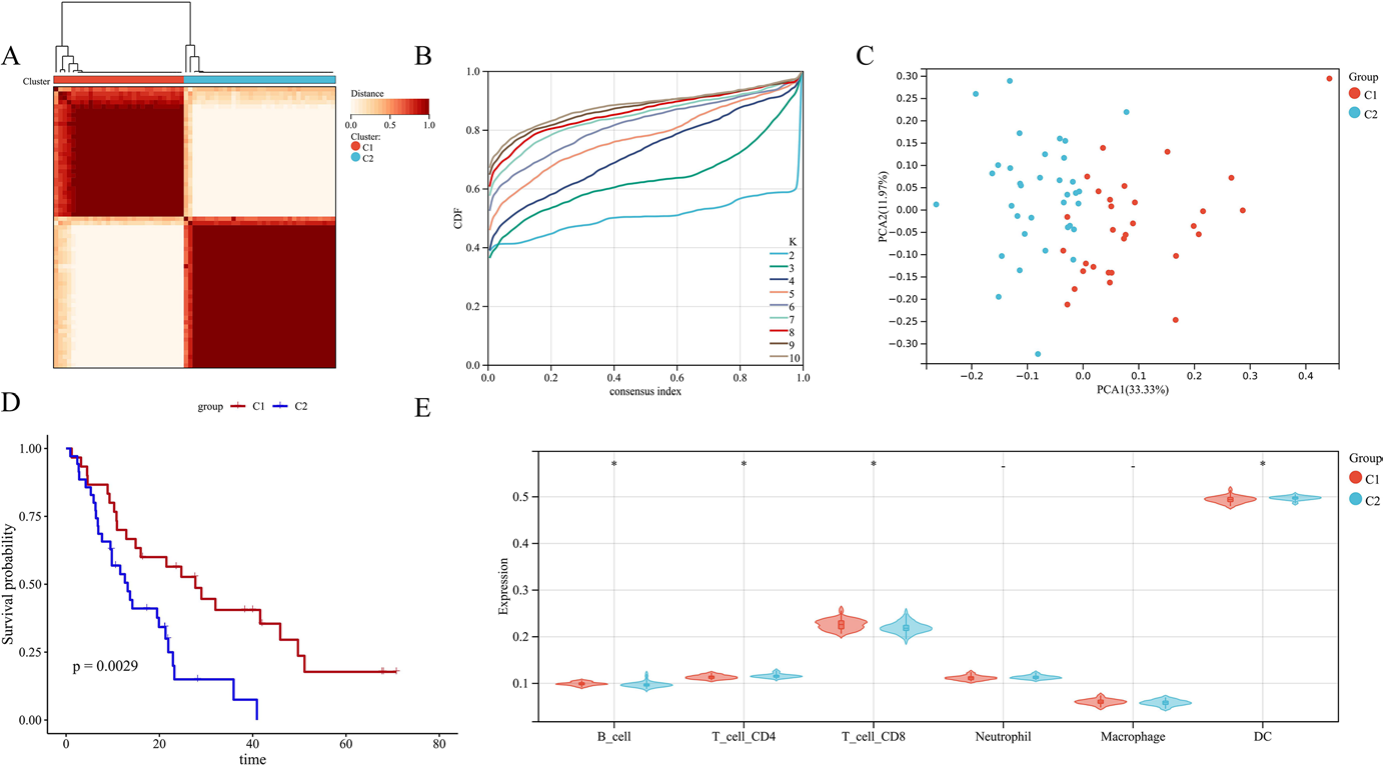
Unsupervised clustering of Lipid metabolism-related genes. **A** Clustering heat map of Lipid metabolism-related genes, 65 samples of GSE62452 were divided into 2 groups (*K* = 2); **B** Consensus Cumulative Distribution Function (CDF) plot under *k* = 2–10, where the number of k represents the number of groups after unsupervised clustering; **C** Principal component analysis (PCA) results based on 82 genes; **D** Survival of patients in C1 and C2; **E** Immune infiltration analysis result of GSE62452 based on TIMERG

We set sixty-five samples obtained from the GEO database (GSE62452) as training set and assessed the impact of genes connected to lipid metabolism in pancreatic cancer samples through Unsupervised cluster analysis (Fig. 2A). According to the results of the cumulative distribution function (CDF) curves and the relative change in area under the CDF curve (Gauci et al. 2013), *K* = 2 was determined as the best cluster number (Fig. 2B). At this time, the C1 and C2 groups consisted of 31 and 34 patients, respectively. This was then validated by PCA, which showed a clear difference between the C1 and C2 groups (Fig. 2C). Therefore, GSE62452 pancreatic cancer specimens were separated into C1 and C2. Kaplan–Meier (Keckesova et al. 2017) curve manifested the significantly better survival rate in C1 (Fig. 2D). Some studies have shown that the upregulation of lipid metabolism function promotes the synthesis of lipids in cell membranes and promotes the rapid development of tumor (Bian et al. 2021).

The immunosuppressive tumor microenvironment, bad T-cell infiltration, and inferior mutation burden drive the acquired immune privilege of pancreatic cancer, resulting in its high lethality (Morrison et al. 2018). To further explore the relationship between different lipid metabolism subtypes and the immune microenvironment, we used the TIMER database to conduct an immune infiltration analysis. The result displayed the less stable expression of B cells, neutrophils, and DC cells in C1 (Fig. 2E). And KEGG enrichment analysis was performed for differentially expressed genes between C1 and C2 groups, and GSEA enrichment analysis was also performed for both groups. KEGG results showed that C1 and C2 differentially expressed genes were mainly associated with Protein digestion and absorption, Fat digestion and absorption, Glycerolipid metabolism, Steroid biosynthesis, Glycine, serine and threonine metabolism, and alpha-Linolenic acid metabolism. Analysis of downregulated genes also showed similar results. Therefore, we believe that the difference between C1 and C2 groups is mainly due to downregulated genes. GSEA results show that C1 and C2 are affected by PENTOSE_AND_GLUCURONATE_INTERCONVERSIONS and VALINE_LEUCINE_AND_ISOLEUCINE_DEGRADATION. There are also significant differences in PROPANOATE_METABOLISM. Therefore, we believe that the metabolic state of group C1 is inhibited relative to that of group C2.

We selected TARGET database for validation, and the unsupervised cluster analysis and CDF curve showed that *K* = 2 was the best cluster number. The Kaplan–Meier curve manifested that the survival rate of patients with C1 was significantly better compared with C2 (*p* = 0.02). It is worth noting that the TIMER immune infiltration analysis showed the same results. That is, the expression of B cells, neutrophils, and DC cells in C1 was less stable than that of C2. Some studies have shown that the increase of B cells and neutrophils is conducive to the occurrence and development of tumor (Minici et al. 2020; Nielsen et al. 2021), while a lack of DC cells will lead to the dysfunction of immune surveillance, which could promote the pancreatic cancer progression (Hegde et al. 2020) (Supplementary Fig. 2). We believe that this found could afford novel insight for the diagnosis and treatment in pancreatic cancer.

### Establishment of Lipid Metabolism-Related Characteristic Scoring Model

**Fig. 3:**
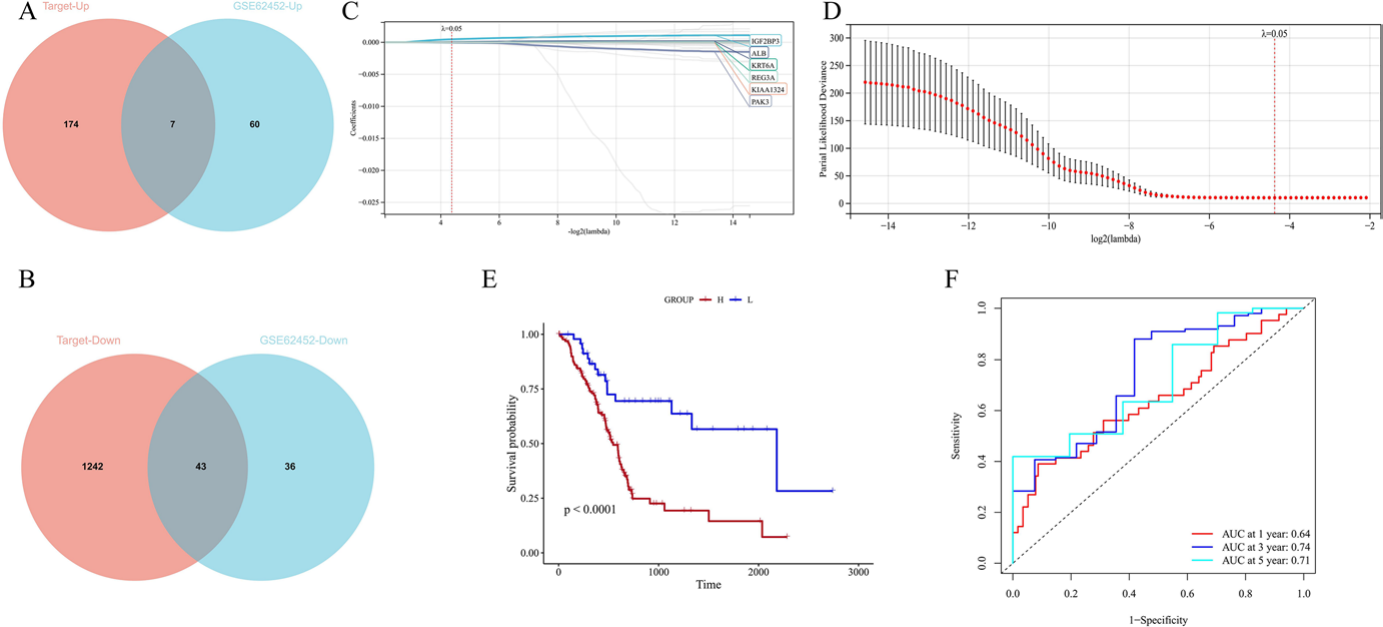
Construction of lipid metabolism characteristic score model; **A** Common upregulated genes of C1 and C2 subtypes in validation set data and training set; **B** Common downregulated genes of C1 and C2 subtypes in validation set data and training set; **C**, **D** Lipid metabolism characteristic score model composed of six candidate genes screened using the LASSO analysis with minimal lambda; **E** Survival difference between high-rating and low-rating groups in the Target cohort; **F** Time-dependent ROC analysis of Target cohort

We used conjoint analysis to research the disparately expressed genes of C1 and C2 in TARGET and GSE62452 databases. The analysis indicated that 43 genes were downregulated, while seven genes were upregulated in C2 compared with C1 (Fig. 3A, B) (supplementary Table 1). LASSO regression analysis was performed, and six genes (IGF2BP3, ALB, KRT6A, REG3A, KIAA1324, and PAK3) were finally selected (Fig. 3C, D).Then, we used GSE62452 to explore the expression of the six genes we finally screened in cancer tissue and adjacent tissues, and the results showed that except for IGF2BP3, the remaining five genes had abnormal expression (supplementary Table 1). Then, we constructed a lipid metabolism characteristic scoring model. Studies have shown that IGF2BP3, ALB, KRT6A, and REG3 partake in the pancreatic cancer origin and development (Mancarella and Scotlandi 2019; Fan et al. 2019; Raman et al. 2018; Zhang et al. 2019). KIAA1324 and PAK3 are associated with kidney renal clear cell carcinoma, hepatocellular carcinoma, and other tumors (Deng et al. 2021; Gao et al. 2022), but their effect on pancreatic cancer has not been well researched.

The scores of each pancreatic cancer sample were calculated and the samples were split into two separated groups, high group with 134 patients and low group with 47 patients, depending on the best intercept value 0.0254453452501767. KM curve showed the significantly higher survival rate in low group (*p* = 0.00012) (Fig. 3E). The ROC curve shows that this staging model can judge the risk and predict the survival rate in pancreatic cancer patients (AUC values of three-year and five-year survival are 0.74 and 0.71, respectively) (Fig. 3F).

### Independence Inspection

**Fig. 4:**
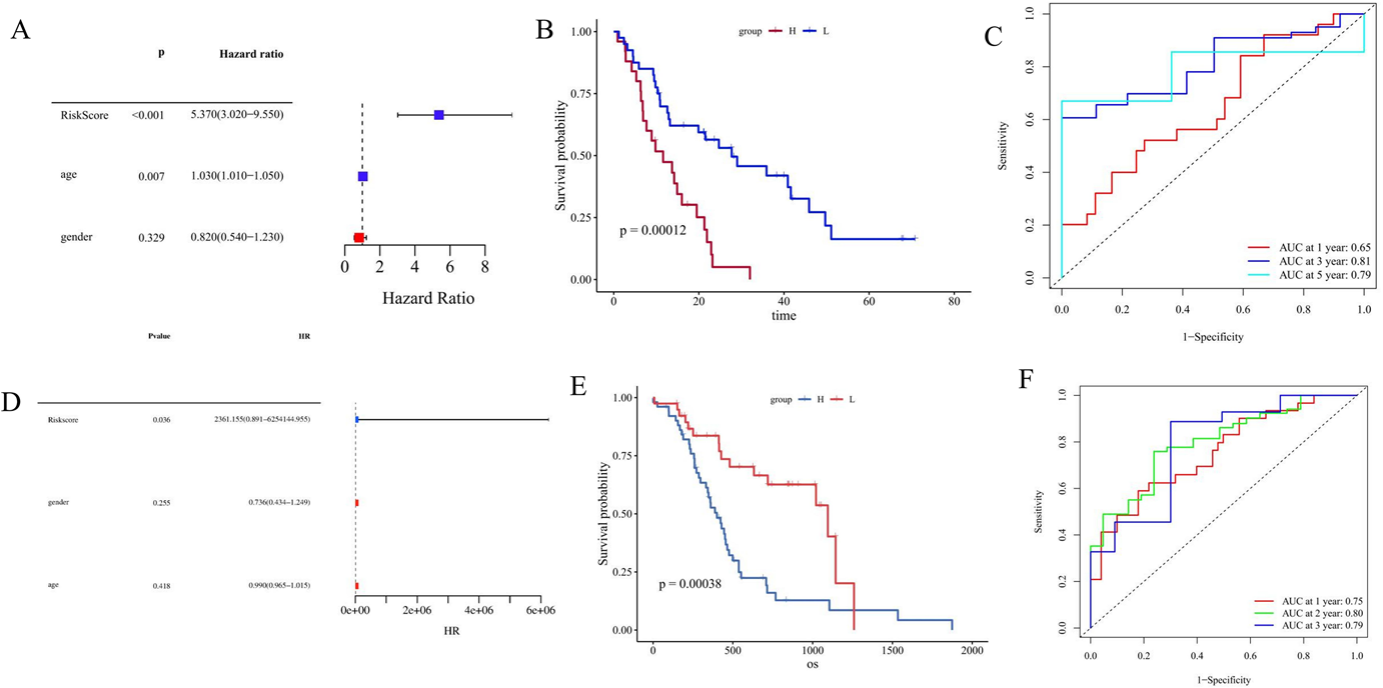
Multi dataset validation of the impact of lipid metabolism-related characteristics on prognosis. **A** Univariate regression analysis to verify the influence of lipid metabolism-related characteristics on prognosis in TARGET; **B** Survival difference between high-rating and low-rating groups in the GSE62452; **C** Time-dependent ROC analysis of GSE62452; **D** Univariate regression analysis to verify the influence of lipid metabolism-related characteristics on prognosis in ICGA.; **E** Survival difference between high-rating and low-rating groups in the ICGC cohort; **F** Time-dependent ROC analysis of ICGC cohort

To check the accuracy and reliability of the scoring model, we conducted univariate cox regression for the risk score according to the expression of the 6 selected genes and the clinical characteristics (risk score, age, and gender) of TARGET samples to determine their relevance and independence (Fig. 4A). This result indicated that both the risk score and age had an independent impact on pancreatic cancer (*p* < 0.001 and *p* = 0.007, respectively). Then, we set GSE62452 and ICGC datasets as validation sets and got each sample risk score calculated. In the GSE62452 cohort, the KM curve showed that the low group (40 patients) had a significantly higher survival rate then high group (25 patients) (*p* = 0.00012) (Fig. 4B), and the ROC curve showed that the AUC of three- and five-year survival rates were 0.81 and 0.79, respectively (Fig. 4C). Since dataset GSE62452 lacks clinical data, such as gender and age, we did not explore the independence of scores from clinical factors. The same results were seen in the low group of 39 patients and the high group of 51 patients in the ICGC, with one patient excluded due to loss of clinical data (Fig. 4E). The ROC curve showed that the AUC of one-year, three-year, and five-year survival rates were 0.75, 0.80, and 0.79, respectively (Fig. 4F). Meanwhile, the results of univariate cox regression analysis showed that risk score could influence the prognosis of patients as an independent factor (one patient was excluded due to loss of clinical data) (Fig. 4D). The previously mentioned results show that our lipid metabolism characteristic score model has substantial predictive value.

### Correlation Between Lipid Metabolism-Related Characteristic Scoring Model and Clinical Characteristics

**Fig. 5:**
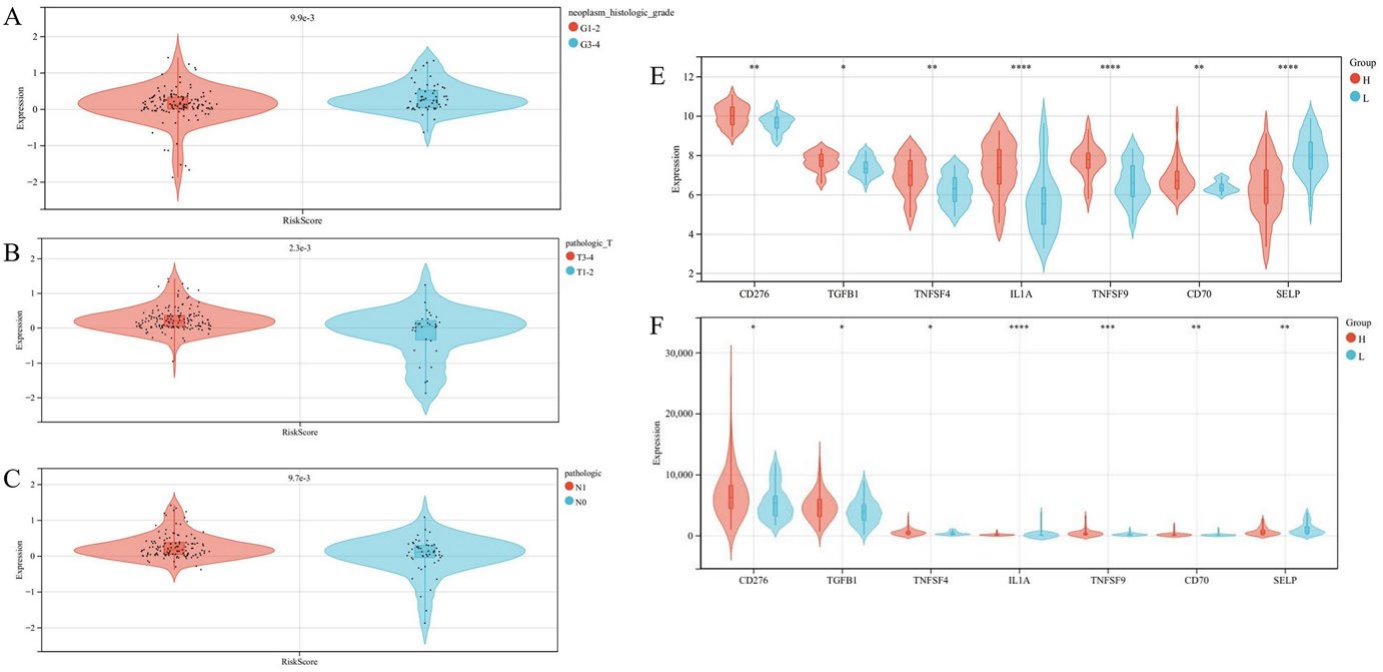
Relationship between lipid metabolism-related characteristics and clinical characteristics. **A** The Relationship between the Characteristics of Lipid Metabolism and Grades (*p* = 9.9 × 10^−3^); **B**, **C** The Relationship between the Characteristics of Lipid Metabolism and T, N Stages (*p* = 2.7 × 10^−3^ and *p* = 9.7 × 10^−3^); **D** Differential expression of seven common immune checkpoint genes based on different model scores in GSE57495; **E** Differential expression of seven common immune checkpoint genes based on different model scores in Target cohort (*, means *p* < 0.05, **, means *p* < 0.01, ***, means *p* < 0.001, ****, means *p* < 0.0001)

To evaluate if our scoring model can judge the clinical staging of pancreatic cancer, we explored its correlation with different stages of pancreatic cancer. The results displayed the significantly worse risk score of G1-2 compared with G3-4 in a general grading system for specific tumor types (Fig. 5A). In terms of TMN score, the risk score of N0 group is below a certain set of N1 group (Fig. 5B) and T1-2 group has an inferior risk score than that of T3-4 group (Fig. 5C). It is evident that the results of our scoring model are highly consistent with clinical data, and it can effectively distinguish between patients with early stages of pancreatic cancer and those with late stages of pancreatic cancer.

### Potential Therapeutic Targets Based on Immune Checkpoints

The immune microenvironment has a close correlation with the survival rate of various tumor patients (Chen et al. 2015; Quail and Joyce 2013). In order to make a further inquiry to explore the expression differences of the six selected genes partaking in the immune microenvironment and immune checkpoints, we conducted a gene differential expression analysis using TARGET as the training set and GSE57495 as the verification set. The previous analysis indicated that the expression of CD272, TGFB1, TNFSF4, IL1A, TNFSF9, and CD70 was upregulated in the high group (Fig. 5D, E). It warrants noting that SELP immune checkpoint gene was downregulated. Some studies have shown that the gene polymorphism of SELP could predict the risk of developing cachexia in pancreatic cancer (Avan et al. 2014). This may provide a potential way for the treatment of pancreatic cancer.

## Discussion

Lipid metabolism is critical for tumor cells. High lipid levels can enhance expression of oncogenic KRAS, resulting in more fibrotic stroma that enhances the tumor progression and stimulates the proliferation of tumor cell lines in pancreatic cancer (29). Our study explores the role of lipid metabolism-related functions in pancreatic cancer, which may guide the clinical immunotherapy of pancreatic cancer. We identified 893 genes closely related to pancreatic cancer lipid metabolism, performed a univariate cox regression using GSE62452, screened out a gene that was not expressed in lipid metabolism in the TARGET database, and finally selected 82 genes. We divided patients with pancreatic cancer into two subtypes C1 and C2 using an unsupervised clustering method, and we used CDF and PCA to verify the rationality of our grouping. To study the differences between subtypes, a six-gene lipid metabolism-related feature scoring model (IGF2BP3, ALB, KRT6A, REG3A, KIAA1324, and PAK3) was established. Then, we investigated the correlation between lipid metabolism scores and pancreatic cancer staging and immunotherapy.

We studied the association of these six genes with tumor, and our scoring model was able to predict pancreatic cancer progression and immunotherapy effect. There are studies that have reported the negative correlation between insulin-like growth factor 2 messenger RNA-binding protein 3 (IGF2BP3) and the prognosis of various tumors, such as bladder tumor prognosis. IGF2BP3 could promote JAK/ Activation of the STAT pathway to inhibit tumor cell apoptosis and furtherance cell proliferation and cell cycle progression. It also exhibits an oncogenic role in human bladder tumor progression (29). IGF2BP3 can inhibit the apoptosis of bladder tumor cells by activating the JAK/STAT pathway. It promotes cell proliferation and cell cycle progression, showing carcinogenic effects in human bladder tumor progression (Huang et al. 2020). It is also highly expressed in patients with liver cancer, and it enhances the invasiveness of hepatoma cells by directly binding to 3′ untranslated region (3′-UTR) and inhibiting the expression of zone occlusion-1 (ZO-1). This results in a poor prognosis for patients (Gao et al. 2020). Serum albumin (ALB) concentration is strongly associated with OS in patients with endometrial cancer. TNF-α, a cytokine known as a mediator of inflammation and tumor-associated cachexia, controls the concentration of ALB. It elevates and promotes uterine tumor invasion and metastasis. The combined action of ALB and TNF-α affects tumor progression and improves patient survival. Research shows the association between keratin 6A (KRT6A) and the progression of various tumors. KRT6A is differentially expressed in pancreatic duct adenocarcinoma (PDAC) and adjacent normal tissue (ANT), which can mediate tumor-associated macrophage subtypes in PDAC changes in β-catenin. β-catenin cascade can induce tumor formation through ErbB2 and make cells cancerous. And this indicates that high concentrations of KRT6A serum albumin (ALB) are closely related to the occurrence and development of OS in patients with endometrial cancer (Zhang et al. 2020). Islet-derived protein 3A (Reg3A), a kind of protein in the REG protein family, is known as liver tumor-gut-pancreas-associated protein or human pancreatitis-associated protein. Reg3A is considered an oncogene in various tumors. It is overexpressed in gastric cancer cells and influences the proliferation, migration, invasion and adhesion of gastric cancer cells by regulating the JAK2/STAT3 signaling pathway accelerating the progression of gastric cancer (Chen et al. 2017). Activation of the Reg3A gene alters the JAK2/STAT3 pathway in pancreatic cancer cells, which can increase the expression of REG3A gene, forming a positive feedback mechanism to further promote the expression of REG3A in tumor cells. This Self-induction of REG3A enhances its tumorigenicity (Guo et al. 2021). This indicates that REG3A can promote tumor progression. Insulin inhibitory receptor (KIAA1324) is negatively associated with the progression of various tumors. For example, it has adverse effect on proliferation, invasion, and drug resistance of tumor cells in gastric cancer and induces apoptosis by inhibiting the activity of GRP78 oncoprotein. This plays an important role in the treatment and diagnosis of gastric cancer patients. It can also be involved in tumor progression and metastasis through two novel splice variants of its mRNA, possibly through its expression level or variable expression of alternative splice products (Bauer et al. 2004). There is a member of the PAK family, P21-activated kinase 3 (PAK3), a serine or threonine protein kinase, that could achieve Epithelial–mesenchymal transition (EMT) of hepatocellular carcinoma by regulating Smad2 and Smad3. Proliferation, metastasis, and invasion worsen the development of hepatocellular carcinoma. Moreover, PAK3 gene expression is higher in hepatocellular carcinoma patients and has positive correlation with tumor stage and grade (Gao et al. 2022). Our study further illustrates that IGF2BP3, ALB, KRT6A, REG3A, KIAA1324, and PAK3 are of great significance in lipid metabolism.

Existing samples were divided into two groups: high group and low group via median-risk score. The survival rate of the high group was significantly lower than that of the low group, which was beneficial for studying the relationship between lipid metabolism and tumor stage and immunotherapy. Next, for evaluating the clinical applicability of the six-gene pancreatic cancer staging model, we used GSE62452 and IGGC as the validation set to perform risk score analysis and found that the survival rates of both groups vary. The ROC results were as follows: the AUC for one-year, three-year, and five-year survival were 0.65, 0.81, and 0.79, respectively. In the TARGET cohort, the AUC for one-year, three-year, and five-year survival were 0.75, 0.80, and 0.79, respectively. The previously mentioned results are consistent with the results of the training set scoring model. Therefore, we concluded that there is a significant association between the high lipid metabolism characteristic score and the poor prognosis in pancreatic cancer patients, indicating that our scoring model can predict prognosis. We explored differences in lipid metabolism signature scores in different stages of pancreatic cancer and found that, in a general grading system for specific tumor types, the G1-2 group has an inferior risk score than that of the G3-4 group. In terms of TMN score, the risk score of N0 group below a certain set of N1 group and T1-2 group has an inferior risk score than that of T3-4 group. This shows that high lipid metabolism score often predicts the advanced development of pancreatic cancer. Therefore, our scoring model could help judge tumor staging in clinical pancreatic cancer patients by providing a more precious and convenient diagnostic reference for the clinical diagnosis and therapy of pancreatic cancer patients.

Additionally, the development of tumors is inseparable from the tumor microenvironment. Therefore, to further pursue the intricate relationship between the scores and the immune microenvironment of pancreatic cancer, we performed a series of immune-related studies and ensued the significant differences in B cells, neutrophils, and DC cells in patients with pancreatic cancer. Transcriptional regulator BCL6 in B cells disrupts their differentiation into antitumor plasma cells and reverses dysfunctional B-cell differentiation. Simultaneously, BCL6 could facilitate the accumulation of intratumoral plasma T cells and effector T cells. Besides, B cells could produce IL-35 in tumor regulates the unique transcriptional state of B cells and antagonizes plasma cell differentiation by stably expressing B-cell lineage-defining transcription factors Pax5 and Bcl6, to enable pancreatic tumor growth (Mirlekar et al. 2022). Myeloid-derived suppressor cells (MDSCs) in neutrophils. These neutrophil-derived MDSCs not only attenuate the antitumor activity of tumor-infiltrating lymphocytes but also promote tumor activity by producing a broad range of mediators. Development of adjunctive strategies for the recruitment and/or deleterious activities of its immunosuppressive mediators (Rapoport et al. 2020). There is a type of antigen-presenting cells (APCs), Dendritic cells (DCs) that could inhibit primary tumors and cause tumor regression by producing a broad range of mediators that activate IL-1 and TNF-α genes. Additionally, we investigated the relationship between lipid metabolism signature scores and seven common immunotherapy targets. For patients with high lipid metabolism scores, the expression of CD272, TGFB1, TNFSF4, IL1A, TNFSF9, and CD70 was higher, while the expression of the SELP immune checkpoint gene was lower. This suggests that it may be a boon for the clinical therapy for pancreatic cancer patients.

In this study, T_cell_CD8 infiltration showed high levels in group C1, while T_cell_CD4 infiltration showed high levels in C2. We know that naive CD4 + T cells can differentiate into helper T cells with different functions according to different cytokines. CD4 + Th1 cells, stimulated by IL-12, IFN-γ, and IL-2, secrete pro-inflammatory cytokines, and have anti-cancer effects (Quail and Joyce 2013). There was no difference in CD4 + Th1 cell infiltration between the two cancer subtypes, but CD4 + Th2-cell infiltration was significantly increased in C2. At the same time, CD4 + T-cell infiltration was significantly higher in C2. This may lead to further development of CD4 + Th2-cell infiltration, which promotes the development of the cancer, resulting in its poor survival rate. DCs are central regulatory factors for adaptive immune responses and are therefore essential for T cell-mediated cancer immunity. DC maturation is necessary to provide co-stimulatory signals to T cells. However, although DC maturation occurs within cancers, considering the inhibitory mechanisms within the cancer, it is usually not sufficient to induce effective immunity (Gardner and Ruffell 2016). Many molecules found in cancer microenvironment can inhibit DC activation in vitro. This includes vascular endothelial growth factor (VEGF), prostaglandin E2 (PGE2), and IL-10. Additionally, VEGF, IL-6, IL-10, and colony-stimulating factor 1 (CSF-1) have been shown to inhibit maturation of bone marrow progenitors or monocytes into DCs. Metabolic dysfunction within the cancer such as hypoxia and lactate regulation dysfunction can affect the function of macrophages within the cancer (Doedens et al. 2010; Colegio et al. 2014) and inhibit DC activation in vitro (Liang et al. 2015; Gottfried et al. 2006). Due to these complex mechanisms, despite high levels of DC-cell infiltration in C2, DC cells may not be able to activate the immune response effectively and immunosuppression occurs, resulting in poor survival. Based on the above results, the immune microenvironment of cancers is complex, and various factors are not single and absolute for the occurrence and development of cancers, but multiple.

Cell survival depends on energy and metabolism. Cancer cells require tremendous energy and raw materials to support their high proliferation rate. Although angiogenesis is increased in the TME, it is not sufficient to meet the glucose and oxygen demands of cancer cell proliferation. This forces cancer cells to regulate their metabolic patterns. A wide variety of cancer cells exhibit increased affinity for lipids and cholesterol. In addition to being used as an alternative energy source to compensate for energy shortages, lipids (Wen et al. 2017) also participate in biofilm synthesis and activate complex signaling pathways related to cancer cell proliferation and spread. Examples include increased intake of exogenous lipids and lipoproteins and hyperactivated de novo synthesis. This directly contributed to the malignant transformation of cancer cells and abnormal lipid accumulation in TME, which may have contributed to its poor survival (Corn et al. 2020). Because of the complex composition and underlying mechanisms of lipids, the same type of immune cell may respond very differently to changes in lipid metabolism. For example, excess free fatty acids (FFA) inhibit CTL-mediated cancer cell killing (Kleinfeld and Okada 2005). Taken together, the above results suggest that low levels of lipid metabolism are beneficial to cancer initiation and progression, which in turn leads to poor survival, while the effects on immune status are complex and diverse.

## Conclusion

In conclusion, we identified two distinct lipid metabolism-related subtypes contingent on lipid metabolism-correlated genes. A scoring model is established via the analysis of lipid metabolism-related traits, which has a substantial assistance in predicting the prognosis, staging, determining immunotherapy targets, and providing theoretical diagnostic references in pancreatic cancer.

### Supplementary Information

Below is the link to the electronic supplementary material.

Supplementary file1 (PDF 51 KB)

Supplementary file2 (PDF 361 KB)

Supplementary file3 (XLSX 30 KB)

## Data Availability

The data used in this article can be found in the GENE EXPRESSION OMNIBUS database (https://www.ncbi.nlm.nih.gov/geo/), the Target database ((https://ocg.cancer.gov/programs/target/data-matrix), and the International Cancer Genome Consortium (https://dcc.icgc.org/); further inquiries can be directed to the corresponding authors.
