# Supplementary figures and images for: Lipid Metabolism-Related Gene Markers Used for Prediction Prognosis, Immune Microenvironment, and Tumor Stage of Pancreatic Cancer
Source: Biochem Genet. 2023 Jul 28;62(2):931–49. doi: 10.1007/s10528-023-10457-y (PMC11031448; doi:10.1007/s10528-023-10457-y)

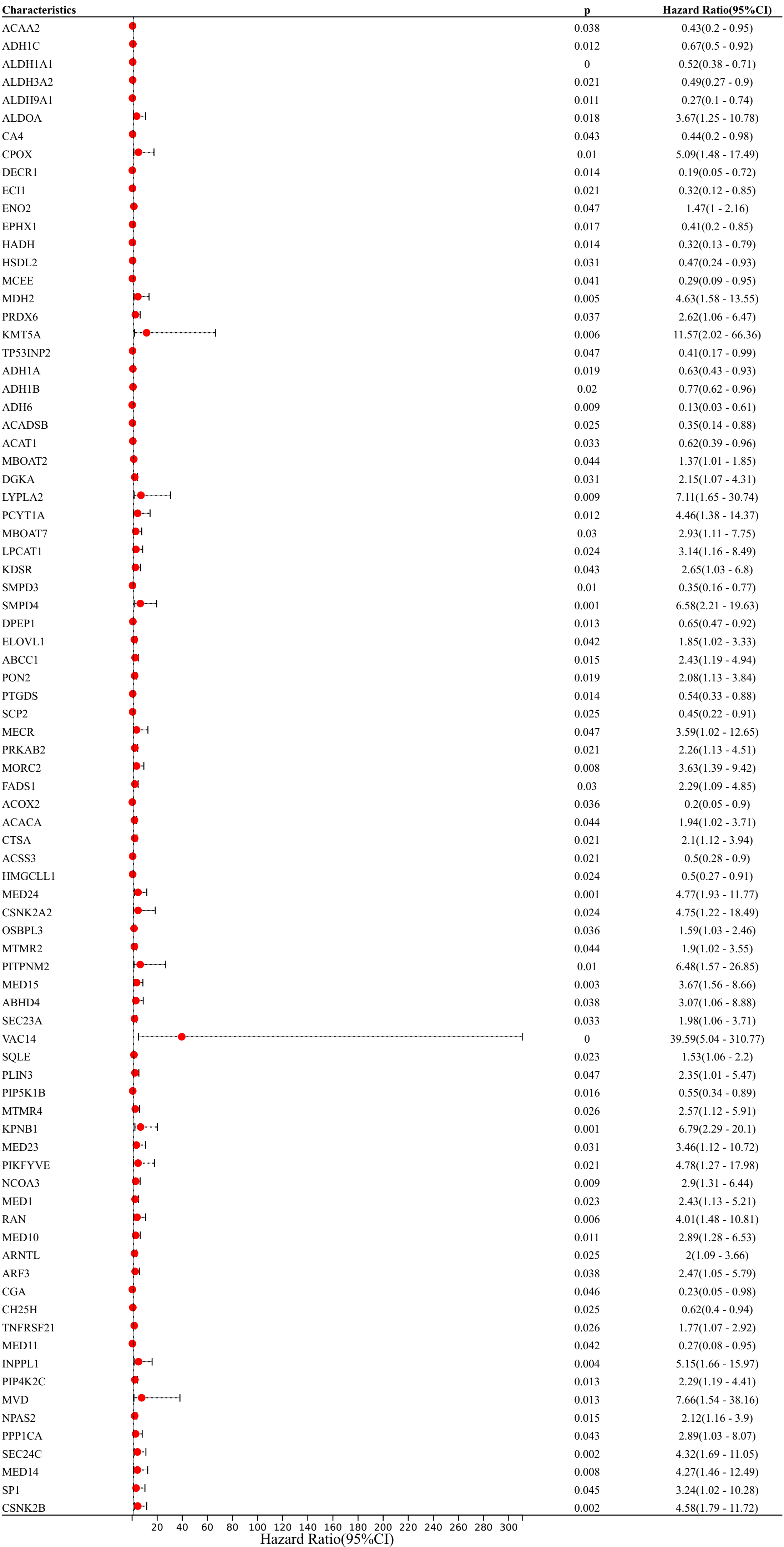

Supplement: Supplementary file 1 — Supplementary file1 (PDF 51 KB) [file 10528_2023_10457_MOESM1_ESM.pdf]

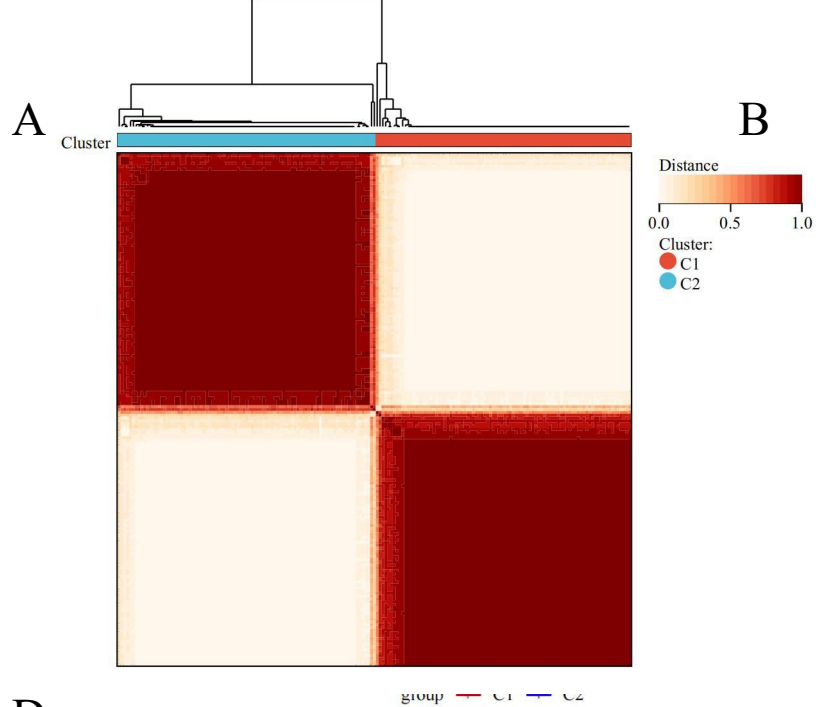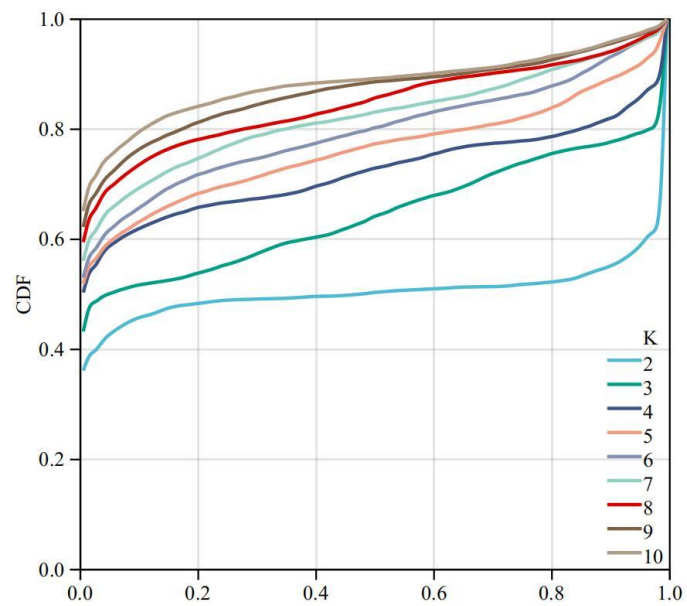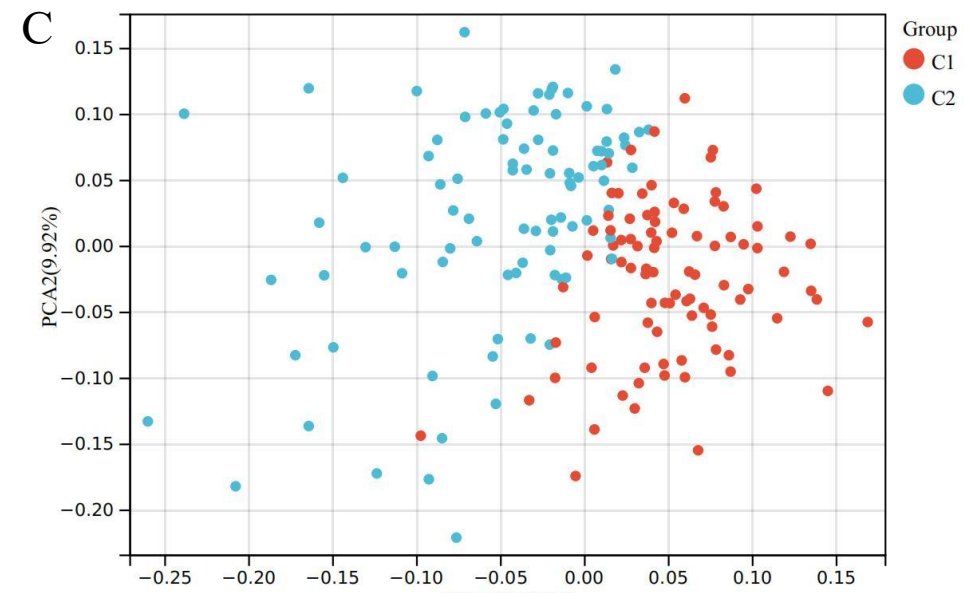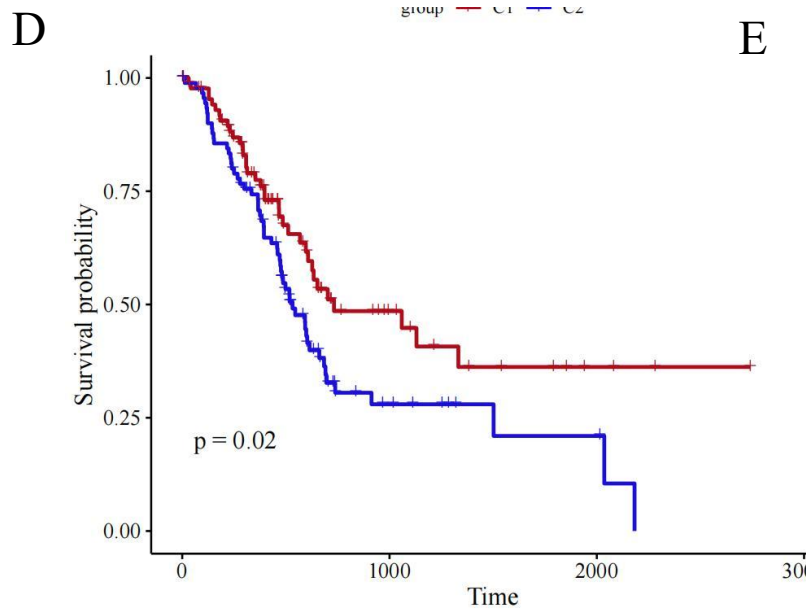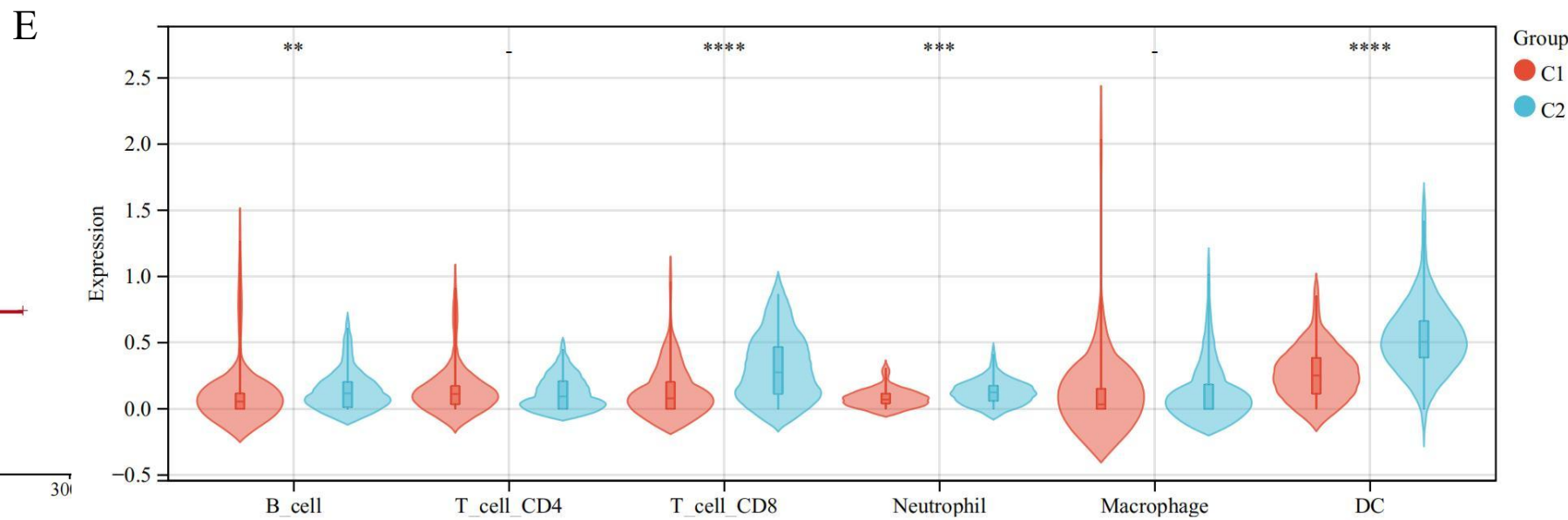

Supplement: Supplementary file 2 — Supplementary file2 (PDF 361 KB) [file 10528_2023_10457_MOESM2_ESM.pdf]
